# Supplementary figures and images for: Morphology of Larger Salivary Glands in Peccaries (Pecari tajacu Linnaeus, 1758)
Source: Animals (Basel). 2024 Oct 8;14(19):2891. doi: 10.3390/ani14192891 (PMC11475750; doi:10.3390/ani14192891)

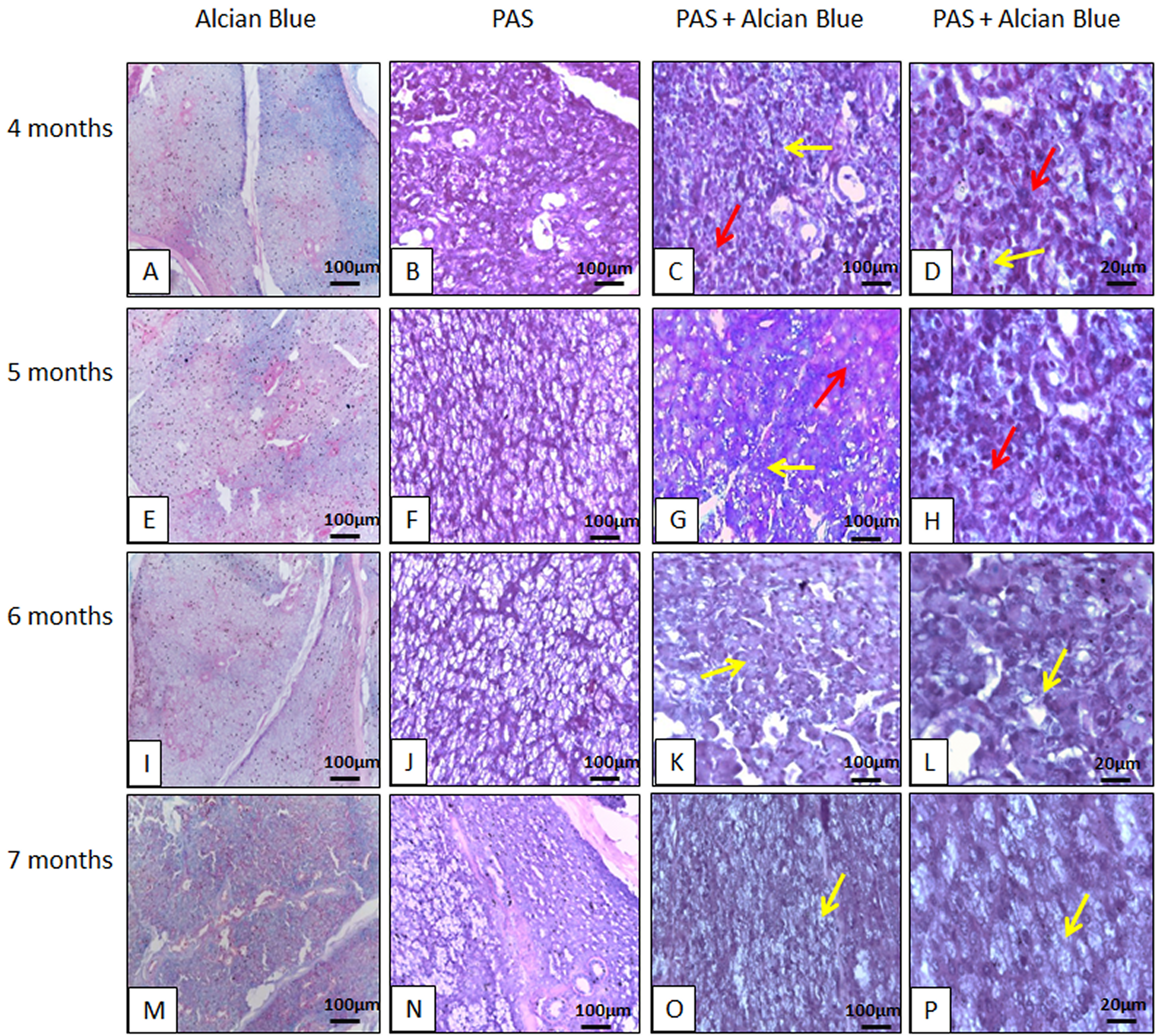

Supplement: Supplementary file 1 [file animals-14-02891-s001.zip › figureS1.tif]

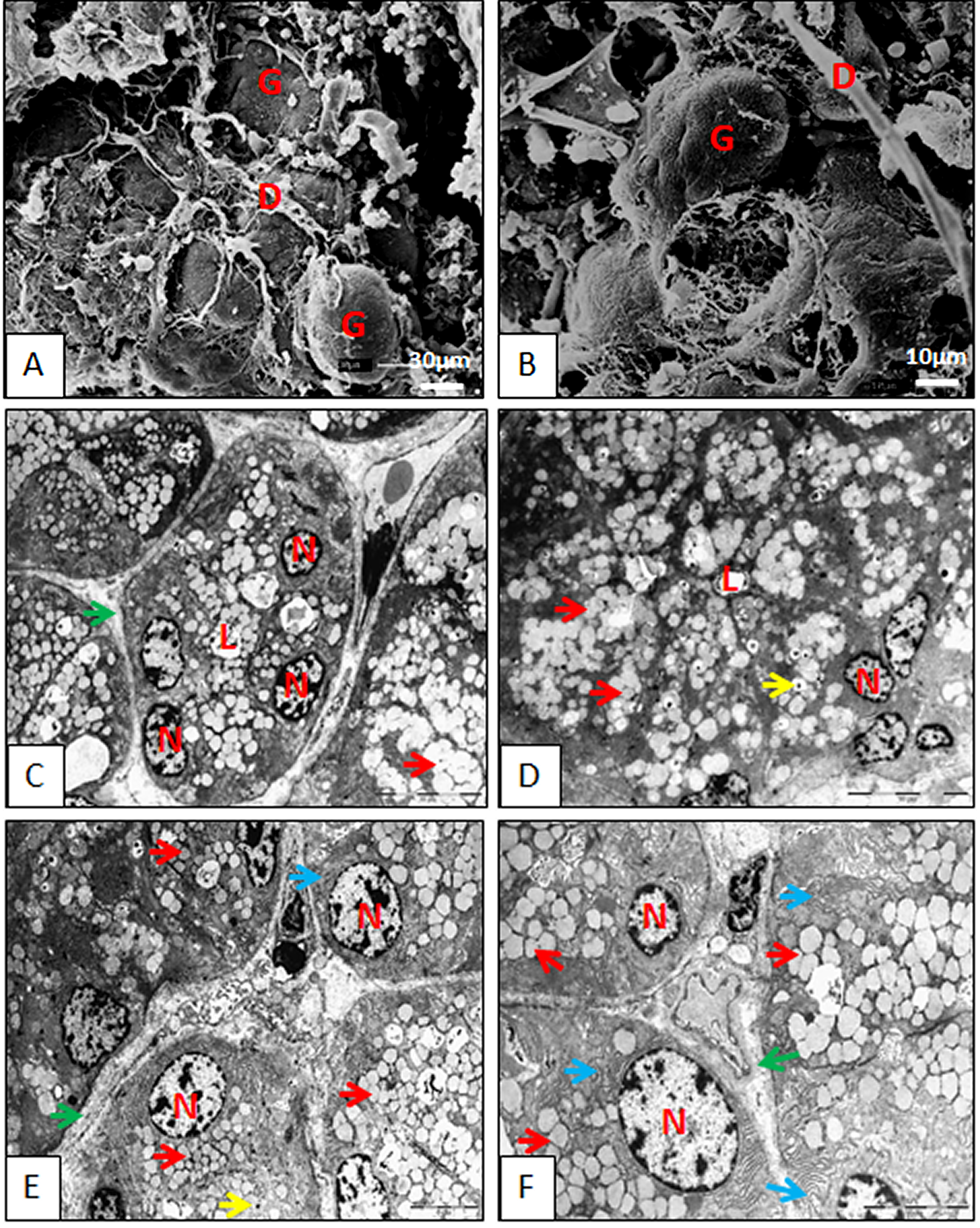

Supplement: Supplementary file 1 [file animals-14-02891-s001.zip › figureS10.tif]

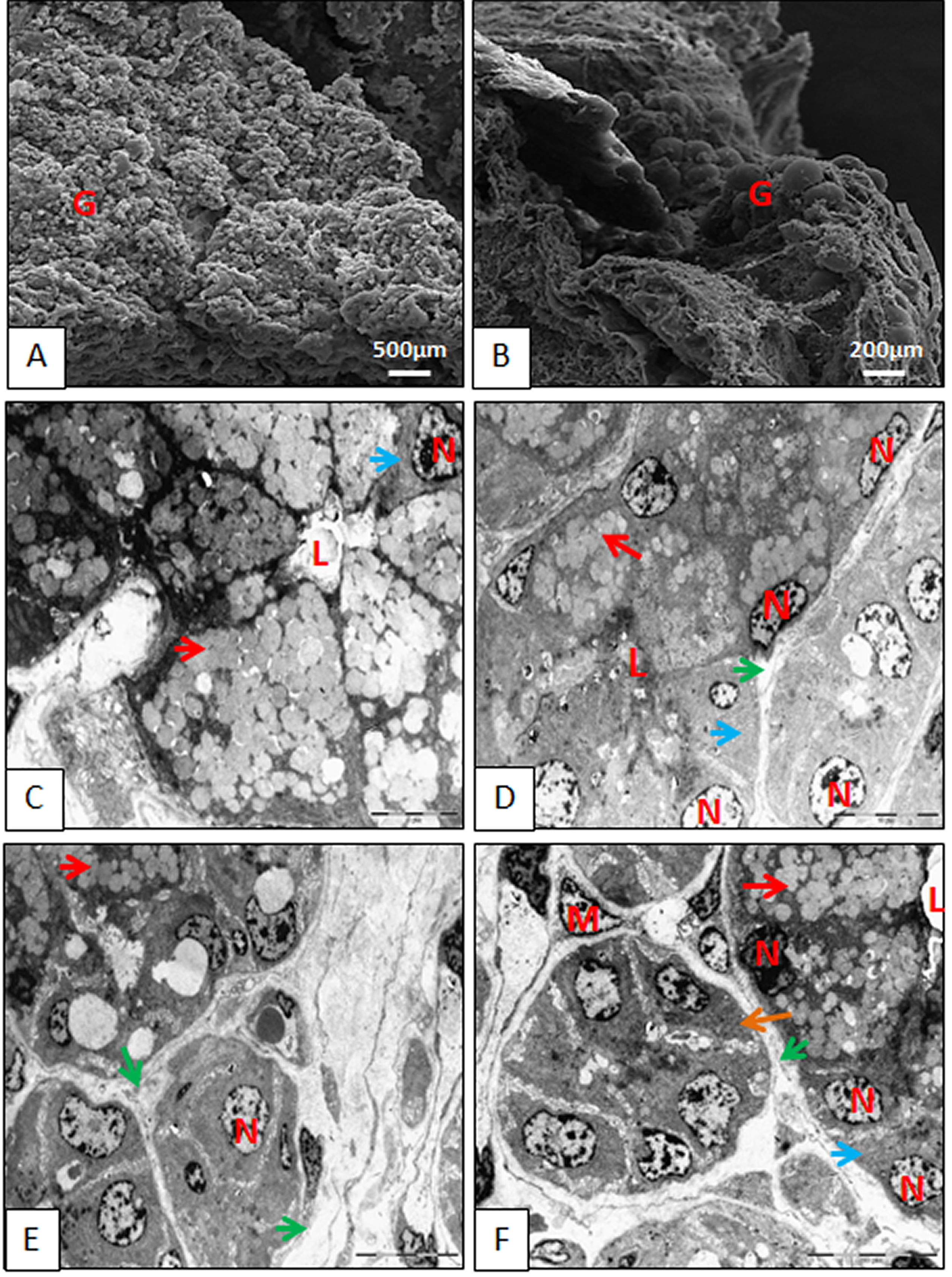

Supplement: Supplementary file 1 [file animals-14-02891-s001.zip › figureS11.tif]

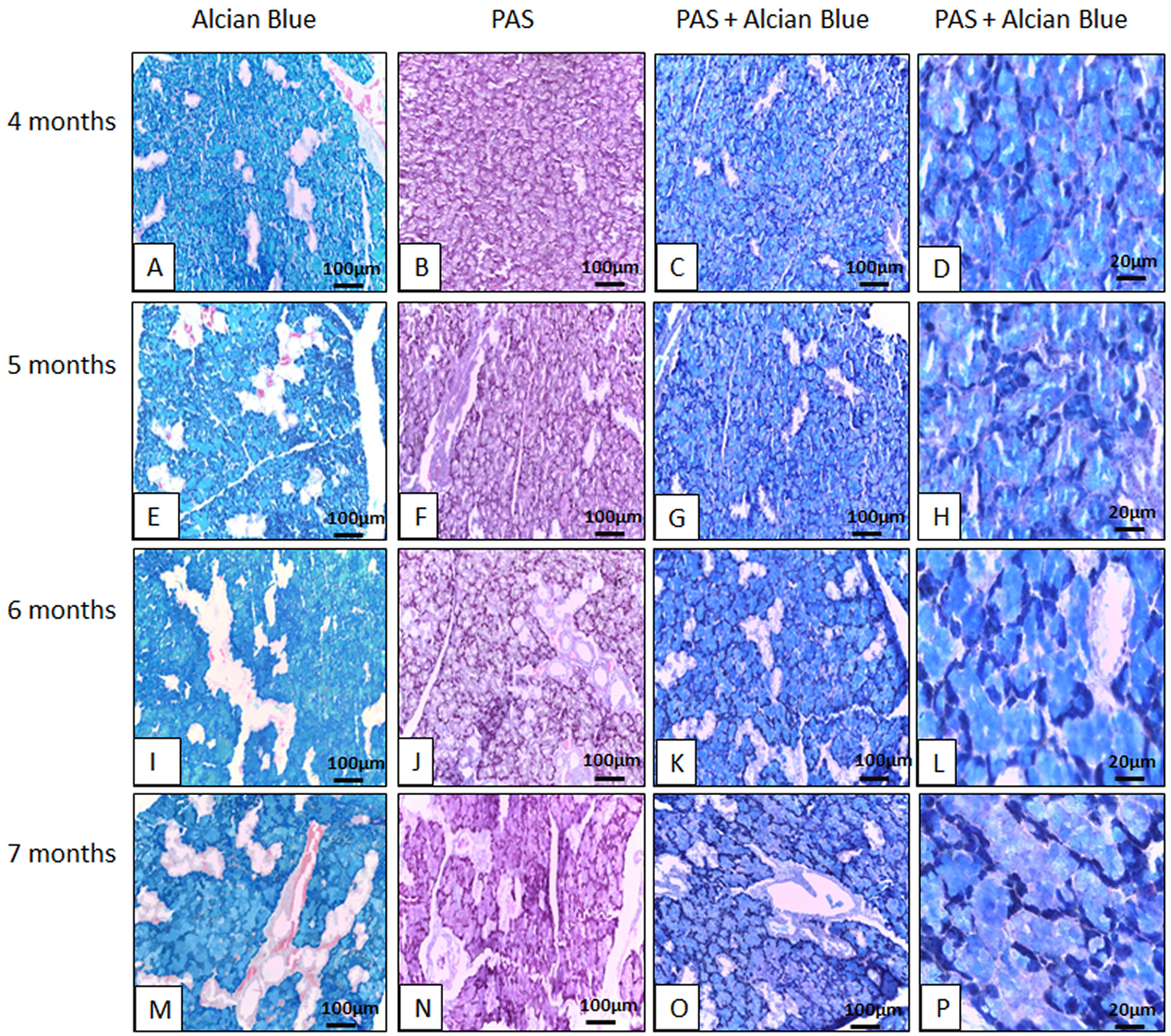

Supplement: Supplementary file 1 [file animals-14-02891-s001.zip › figureS2.tif]

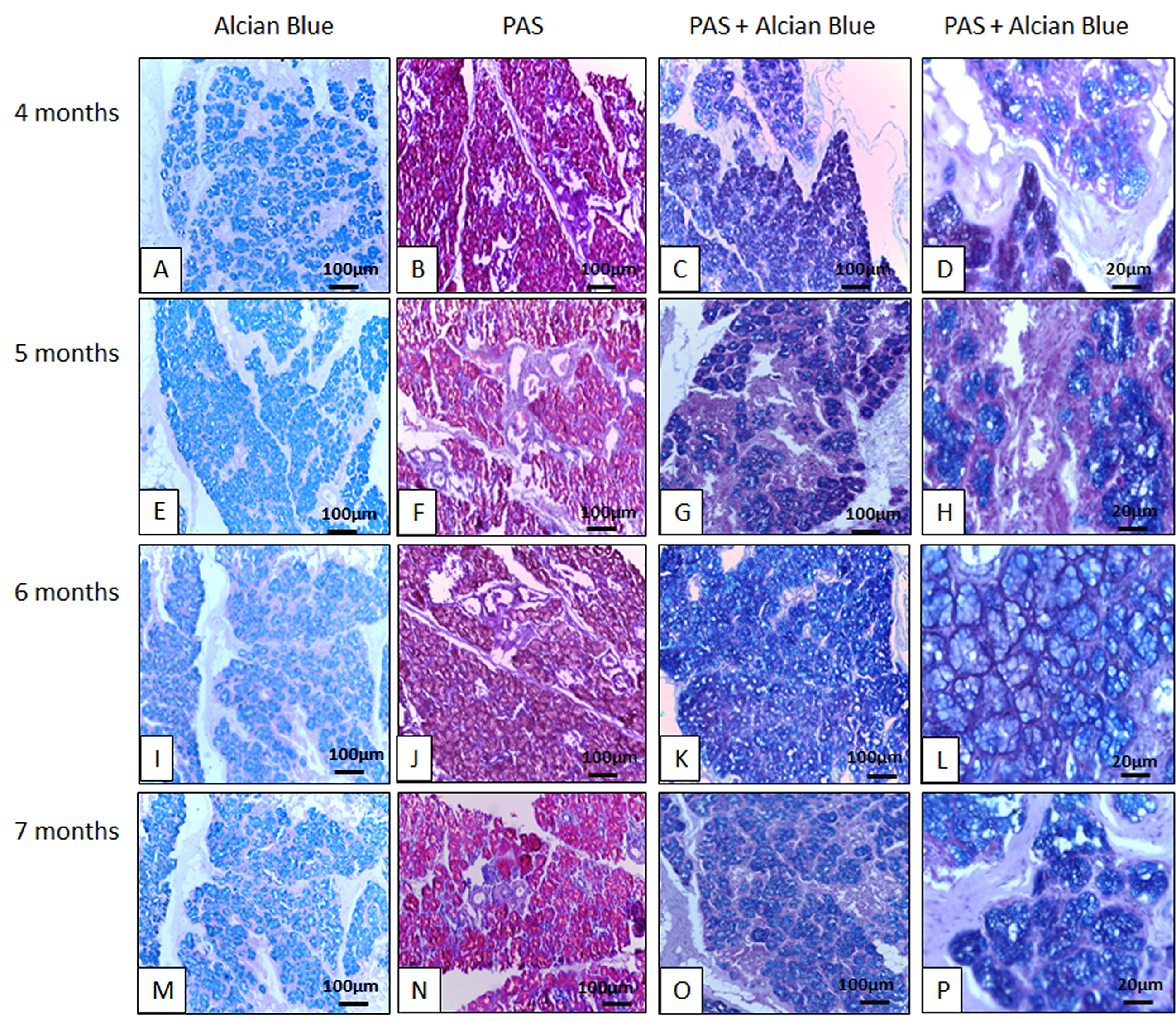

Supplement: Supplementary file 1 [file animals-14-02891-s001.zip › figureS3.tif]

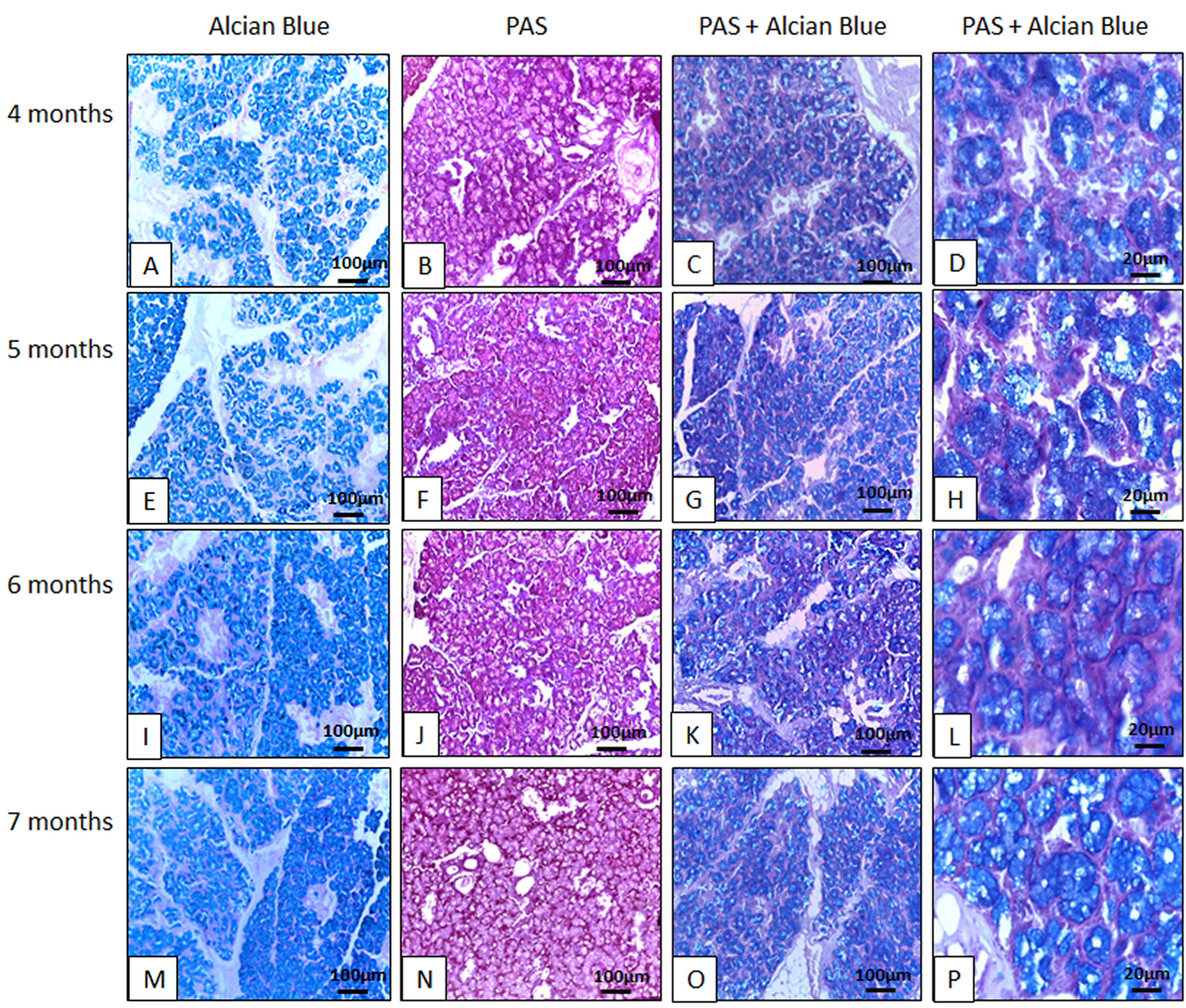

Supplement: Supplementary file 1 [file animals-14-02891-s001.zip › figureS4.tif]

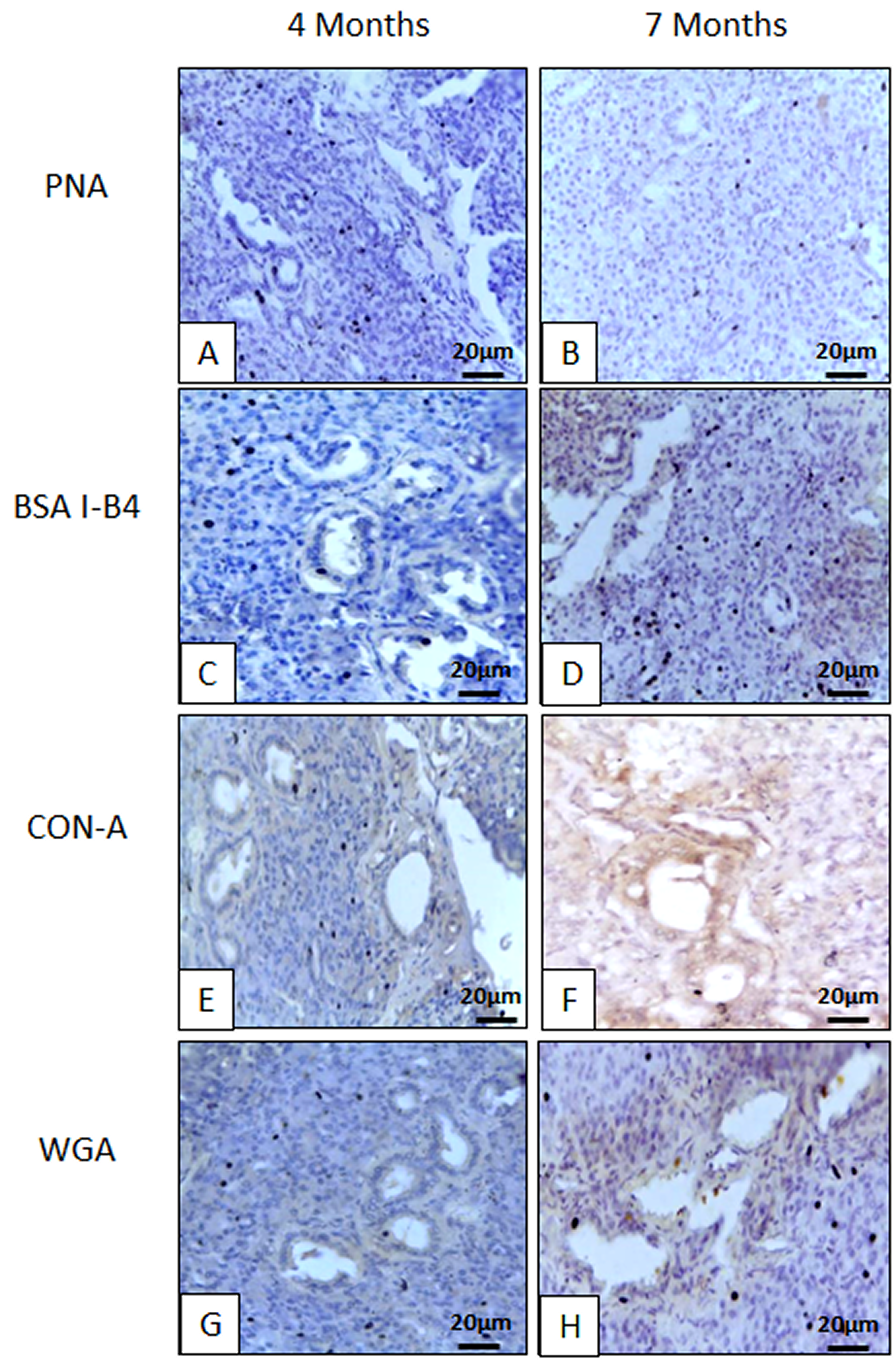

Supplement: Supplementary file 1 [file animals-14-02891-s001.zip › figureS5.tif]

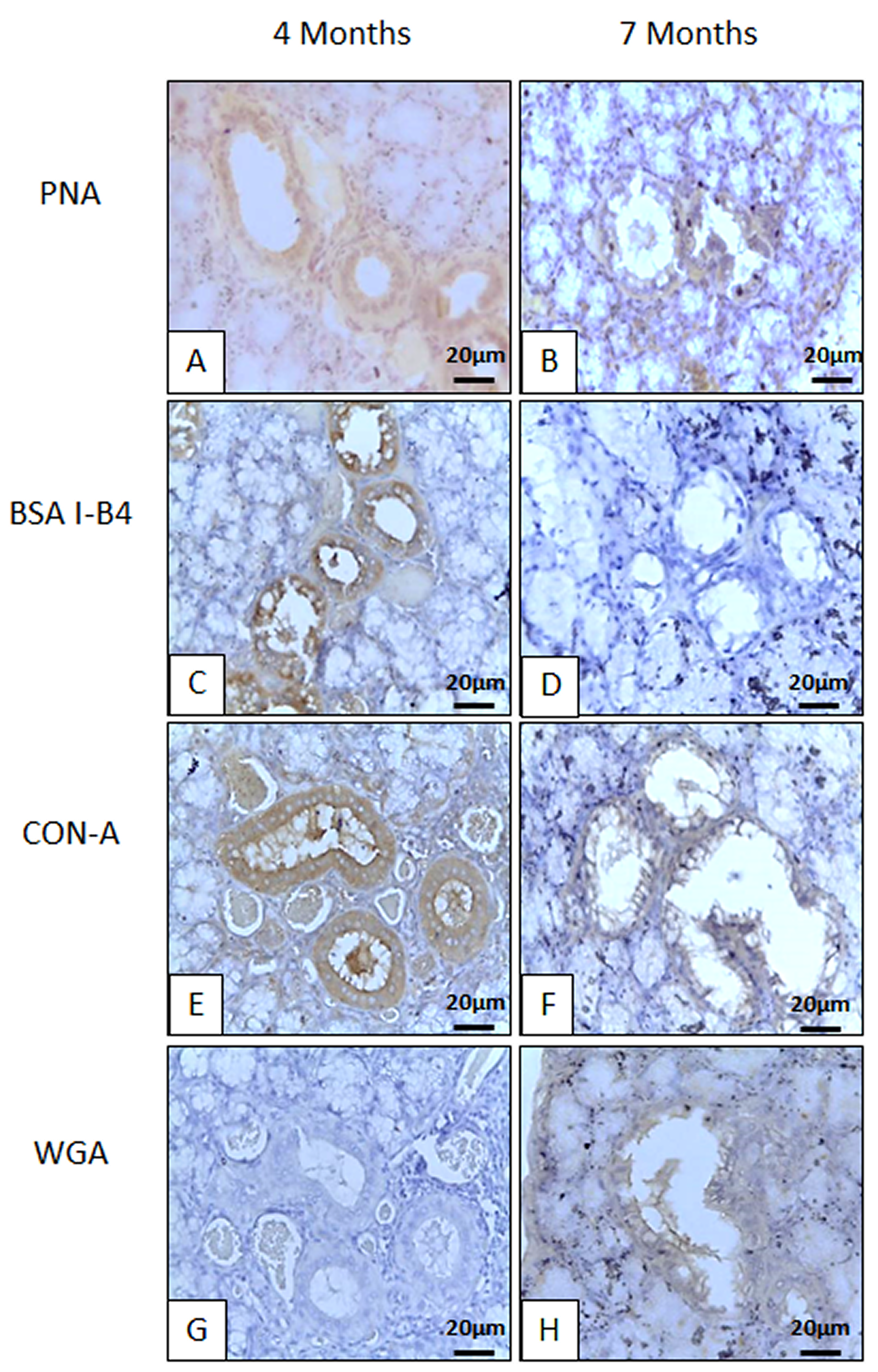

Supplement: Supplementary file 1 [file animals-14-02891-s001.zip › figureS6.tif]

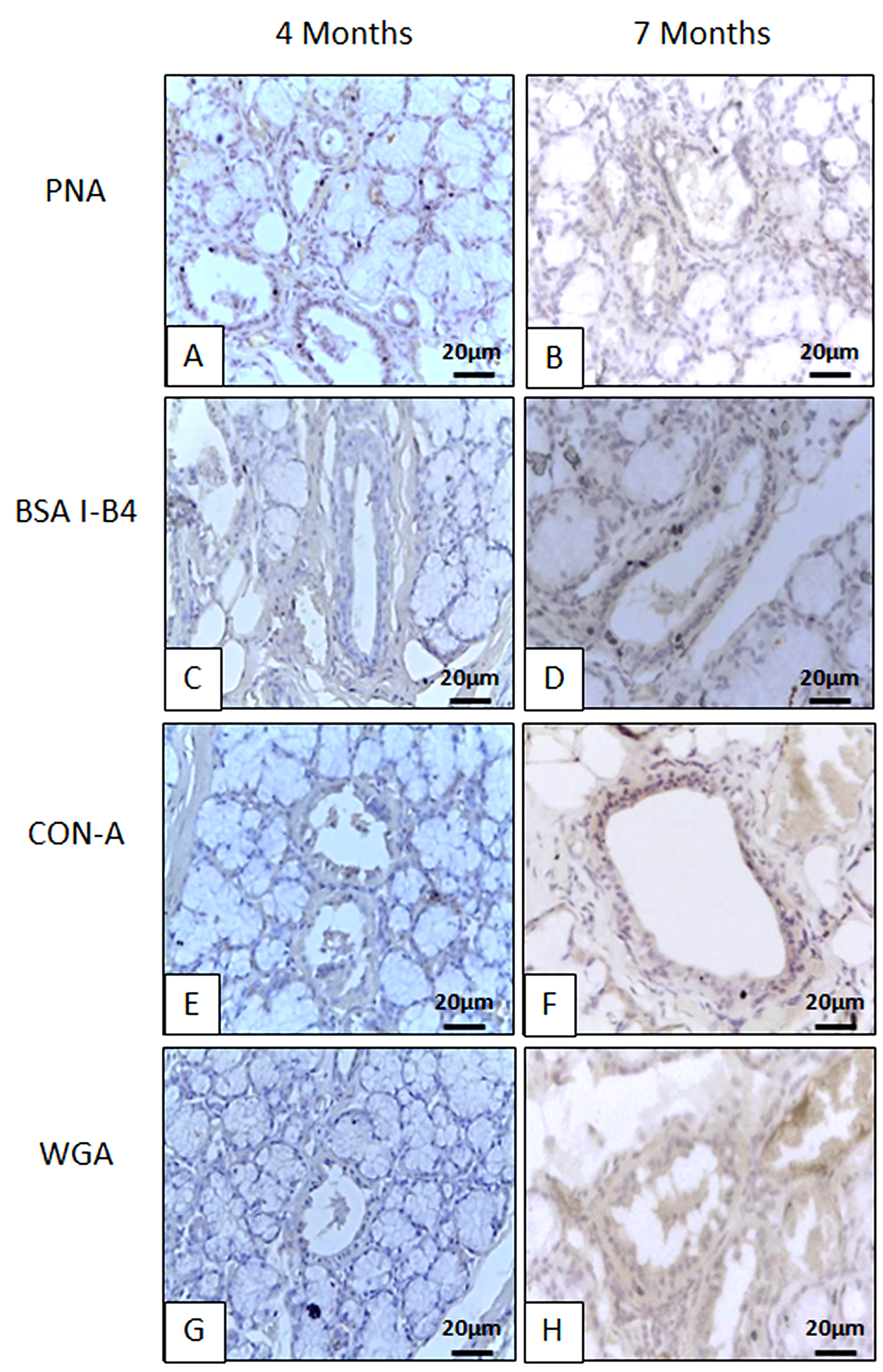

Supplement: Supplementary file 1 [file animals-14-02891-s001.zip › figureS7.tif]

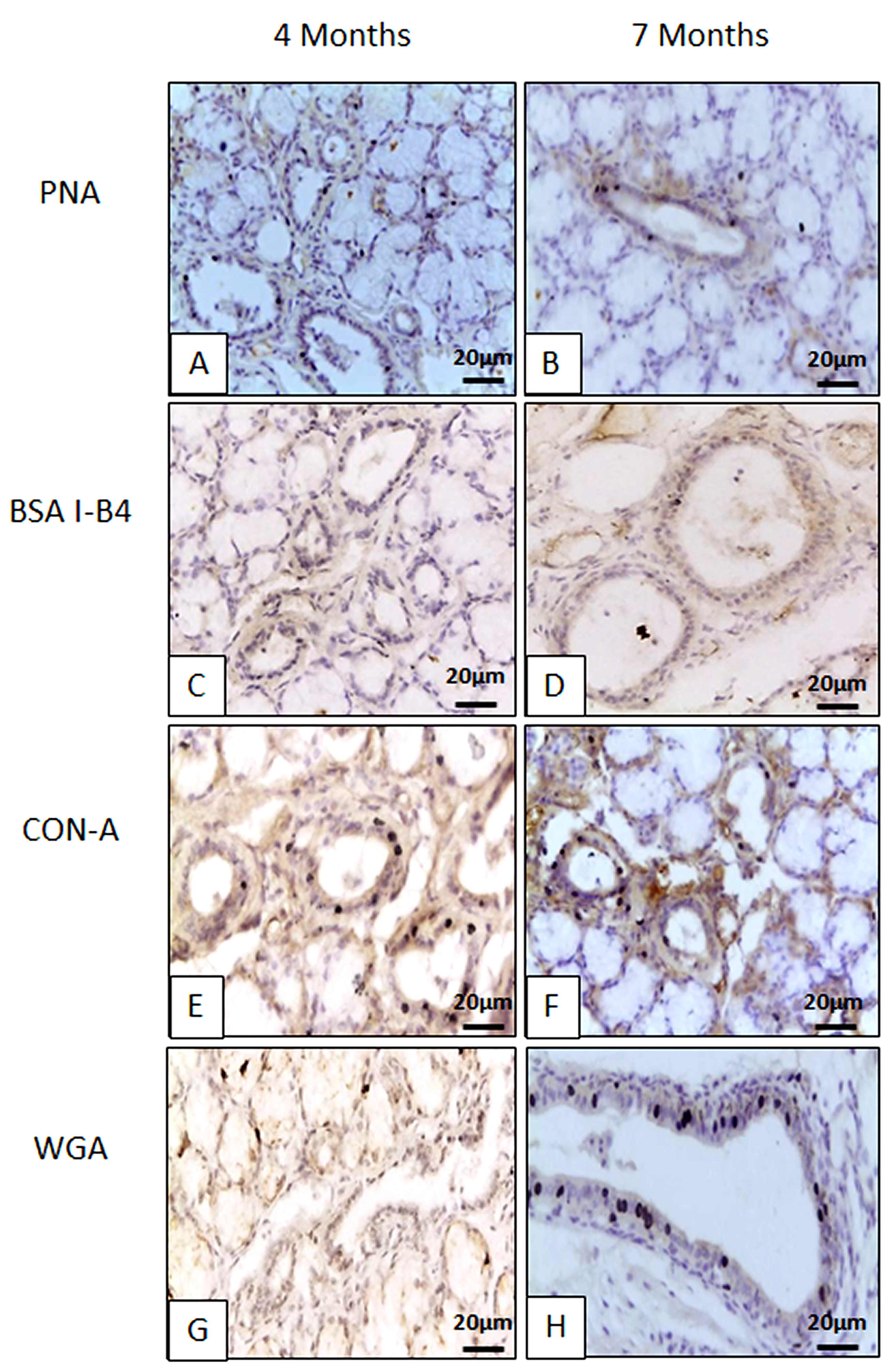

Supplement: Supplementary file 1 [file animals-14-02891-s001.zip › figureS8.tif]

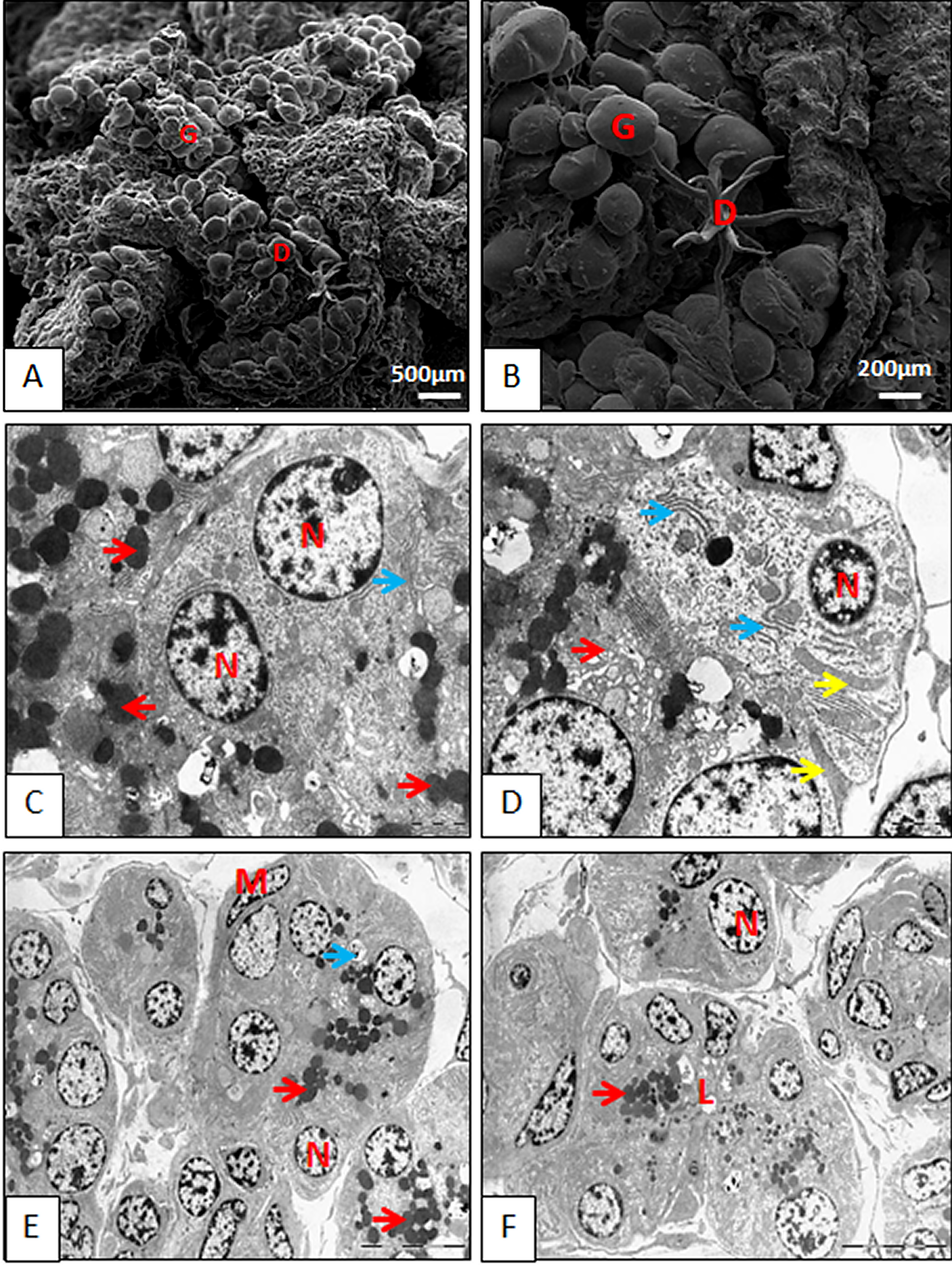

Supplement: Supplementary file 1 [file animals-14-02891-s001.zip › figureS9.tif]
